# Regional Spread of Ebola Virus, West Africa, 2014

**DOI:** 10.3201/eid2103.141845

**Published:** 2015-03

**Authors:** Gabriel Rainisch, Manjunath Shankar, Michael Wellman, Toby Merlin, Martin I. Meltzer

**Affiliations:** Centers for Disease Control and Prevention, Atlanta, Georgia, USA

**Keywords:** Ebola virus, hemorrhagic fever, Ebola, Ebola virus disease, epidemics, models, statistical models, geographic information systems, GIS, spatial analysis

## Abstract

To explain the spread of the 2014 Ebola epidemic in West Africa, and thus help with response planning, we analyzed publicly available data. We found that the risk for infection in an area can be predicted by case counts, population data, and distances between affected and nonaffected areas.

The first cases of the 2014 Ebola epidemic in West Africa (49 cases in Guinea) were reported on March 21 (*1*). By November 4, the World Health Organization had reported 13,241 cases in the 3 primarily stricken countries of Guinea, Sierra Leone, and Liberia and single cases in Senegal and Mali (*2*). Although virus transmission to other countries (Nigeria, United States, and Spain) has occurred via air travel, most infections have spread regionally via ground movement of sick persons. To aid with response planning, we sought to explain this regional spread by assessing publicly available information.

## The Study

The data analyzed were case counts, population data, and distances between affected and nonaffected districts (these distances are influential predictors in the spread of infectious diseases) (*3*–*5*). We first classified as affected those districts within Guinea (prefectures), Sierra Leone (districts), and Liberia (counties) that had reported to the World Health Organization >1 suspected, probable, or confirmed case of Ebola virus infection from the weeks ending March 29, 2014 (epidemiological week 13), through August 16, 2014 (epidemiological week 33) (*2*). For each district, we considered the week of its first reported case as the week it became affected (Technical Appendix Figure 1). We also identified the population-weighted geographic centroid (center of an area, adjusted for its population density) in each district and computed the distance from these centers to similar centers in each affected district.

**Figure 1:**
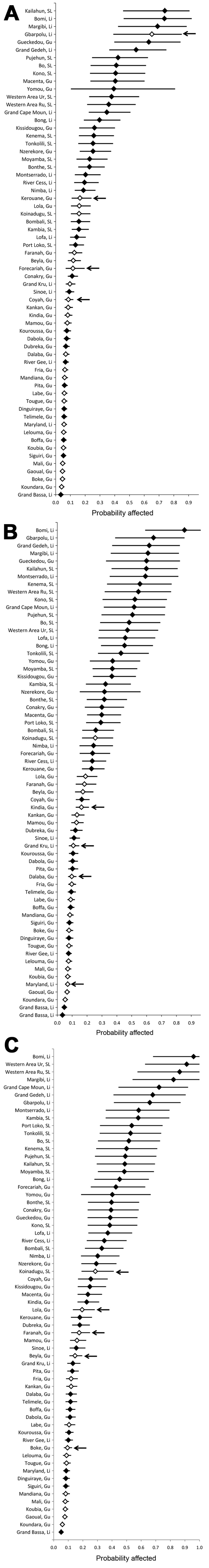
Probability predictions (with 95% CIs) for districts in countries primarily affected by Ebola virus infection in 2014, by week of analysis. A) Data available through week 33 (August 16). B) Data available through week 36 (September 6). C) Data available through week 39 (Sep 27). Diamonds indicate the probability that the districts should be affected at the time of the analysis. Filled diamonds indicate districts that were affected (i.e., had reported at least 1 case) at the date of the analysis. Black arrows identify those districts that became affected within 3 weeks of the date of analysis. SL, Sierra Leone; Gu, Guinea; Li, Liberia.

We then created 4 regression models to calculate the weekly risk of a district being affected as a function of combinations of its population, the sum of inverse distances (SID) from all affected districts, and SID weighted by the number of new cases in affected districts over the preceding 3 weeks (Technical Appendix Table 2). We chose the best model by examining how well it fit the data available through week 33 (August 16). We then evaluated how well the chosen model predicted that districts would become affected as the outbreak continued by comparing calculated probabilities that a district would become affected (at weeks 33, 36, and 39) to actual reports of newly affected districts over the subsequent 3-week periods (weeks 34–36 [period 1], weeks 37–39 [period 2], and weeks 40–42 [period 3], respectively). By using data available through week 42, we calculated probabilities that districts in countries bordering the 3 primarily affected countries (departments in Côte D’Ivoire, circles in Mali, departments in Senegal, sectors in Guinea-Bissau, and divisions in Gambia) would become affected. 

We assumed that country and district borders were porous and that infected persons could not be prevented from moving into nonaffected areas (*6*–*8*). Reports from the field support this assumption, even after country borders were officially closed (*9*). We also assumed no heterogeneities in the capabilities of the different areas to identify and report cases and that aggregating case count reports into a weekly unit of analysis would blunt the effects of reporting delays. Our last assumption, for identifying an affected district, was that suspected and probable cases were as sensitive and specific as confirmed cases.

**Figure 2:**
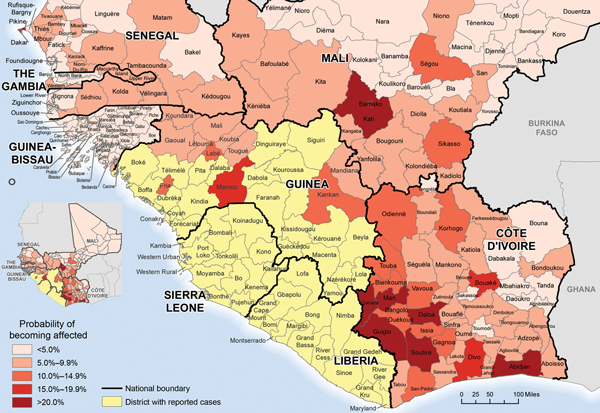
Predicted risk of districts becoming affected by Ebola virus infection (neighboring countries included) in 2014, based on data available through epidemiological week 42 (October 18, 2014).

Among the 3 primarily affected countries, 39 districts were affected in 12 weeks (during weeks 13–33). The model that best explained this pattern was one in which the risk of a district becoming affected depended on its population and the SID from all affected districts to a nonaffected district and in which each inverse distance is multiplied by the sum of new cases within the past 3 weeks (weighted SID) (Technical Appendix Table 2 and Figure 2). The overall average weighted SID was greater for districts during the weeks in which they became affected than for districts that had not yet reported cases by the same week (Technical Appendix Figure 3, panel A).

Figure 1 shows the probabilities for specific districts becoming affected at weeks 33, 36, and 39. The ranking of districts by their probabilities on week 33 (Figure 1, panel A) illustrates the good fit of the model because 27 (87%) of the 31 districts ranked in the top half (most likely to become affected) were actually affected.

During weeks 34–36 (period 1), 4 districts became affected; during weeks 37–39 (period 2), 4 districts became affected; and during weeks 40–42 (period 3), 5 districts became affected. The model predicted well which districts would become affected during periods 1 and 3 (Figure 1, panels A, C); districts that became affected were predominantly among those with the highest calculated probabilities of becoming affected. The model did not predict as well which districts would become affected during period 2 (Figure 1, panel B).

Of 167 districts in the countries bordering the primarily affected countries, the predicted probability of becoming affected was >20% for 9 districts (calculated at week 42). The 3 top-ranked districts had the largest populations in their respective countries: Abidjan (Côte D’Ivoire), Bamako (Mali), and Pikine (Senegal); Bamako and Pikine reported cases in weeks 43 and 35, respectively. Also, among the top 10 districts, 5 were on or near the Côte D’Ivoire–Liberia border (Figure 2).

## Conclusions

We identified spatial influences on the regional spread of Ebola virus infections. The risk of becoming affected by Ebola was significantly higher for nonaffected districts that had a larger population and that were closer to affected districts with higher case counts (Technical Appendix Table 2 and Figure 2). Thus, it seems that data on population size and straight-line distances can serve as pragmatic alternatives to data on travel patterns between Guinea, Liberia, and Sierra Leone during the first 8 months of the outbreak. The correlation between the risk of becoming affected and distances and population size was sufficiently accurate for predicting which districts would next become affected. Furthermore, a high calculated probability of becoming affected for a district considered not affected might indicate the presence of undetected cases.

This analysis relied heavily on the accuracy of case reports and their timely documentation, but there are indications that extreme conditions in the affected countries resulted in incomplete records and reporting delays (*10*). These factors potentially contributed to errors in the identification of which week a district became affected. Consequently, we examined the potential effects of reporting delays (Technical Appendix Table 2). Also, our results might have been influenced by our choice of administrative unit level to use for defining districts. (In our analysis, countries with smaller district units have less risk of being affected than countries with larger district units, if population densities are generally comparable.)

The good fit of our model, absent predictors for the influence of interventions, suggests that interventions (including border closings) were minimally effective at stemming regional spread of Ebola virus infection during the period analyzed. As the spread of the epidemic changes because of interventions and changes in human behavior, there is need to update and reevaluate the model fit and the parameters used.

We chose to not pursue data on travel patterns, despite their potential utility for explaining the spread of Ebola virus infection. Travel patterns may evolve as the outbreak progresses, and obtaining accurate data during an ongoing outbreak is challenging. We, therefore, focused on producing the simplest model.

Overall, our simple model shows that available case reports, population data, and distance data can be used to identify areas at risk of being affected in an outbreak of Ebola virus infection. Additionally, if the current pattern of spread in this outbreak continues, or if the outbreak takes hold in new countries, this model can be used to advocate for allocation of surveillance and control resources to nonaffected areas.

Technical Appendix Additional details on methods and results of study of regional spread of Ebola virus, West Africa, 2014.
