# Supplementary material for: Regional Spread of Ebola Virus, West Africa, 2014
Source: Emerg Infect Dis. 2015 Mar;21(3):444–7. doi: 10.3201/eid2103.141845 (PMC4344291; doi:10.3201/eid2103.141845)
Supplement: Technical Appendix — Additional details on methods and results of study of regional spread of Ebola virus, West Africa, 2014. [file 14-1845-Techapp-s1.pdf]

Article DOI: http://dx.doi.org/10.3201/eid2103.141845

Regional Spread of Ebola Virus, 2014

Technical Appendix

This Technical Appendix provides further details on the methods used as well as additional results.

Data and Definitions

Case Count Data: We obtained the cumulative number of confirmed, probable and suspect case counts for each of the 63 districts in Liberia, Sierra Leone, and Guinea from WHO Situation Reports posted weekly on the WHO's website (*1*). We defined a district as having become affected if it had least one suspect, probable, or confirmed Ebola case in the WHO reports. We considered the week a district first reported a case as the week it became "affected". We also used the case counts data from the WHO Situation Reports in our calculations of the weighted sum of inverse distances (see Calculations section below). We first identified the number of "new cases" in a single given week by subtracting the cumulative case count for a district in a given week from the cumulative case count reported for the week prior. We then summed the new cases values for every three week period in our outbreak dataset to obtain the number of new cases over the prior three weeks.

For some districts, defining the week a district became affected and calculating new cases was complicated by reductions in the cumulative case count from week to week or gaps in reporting. Technical Appendix Table 1 describes how case counts data were used to define the week of first report (i.e. affected) and for case count weighting in these special circumstances.

Other studies (*2–4*) examining the role of distance as a predictor of disease spread used confirmed cases only to determine when a new area had became affected. We did not rely on confirmed cases alone due to heterogeneity in the reporting delay of confirmed cases reported by country (*5*). For example, in Guinea (using data through week 33), the reporting of confirmed cases across all affected districts occurred on just 4 different weeks while the reporting of cases based on all types (i.e. including suspect and probable cases too) was spread across 8 weeks. Reports from the field indicated that this was the result of laboratory confirmation testing occurring via batch processing.

Geospatial Data: We obtained "district" and international boundary data from the GADM database of Global Administrative Areas, version 2.0 (http://www.gadm.org) and from the United Nations Mission in Liberia and the United Nations World Food Program via the United Nations Office

for the Coordination of Humanitarian Affairs' Humanitarian Response website (http://www.humanitarianresponse.info/applications/data). Districts were defined for the primarily affected countries as the equivalent of Prefectures in Guinea, Districts in Sierra Leone, and Counties in Liberia. For bordering countries, districts were defined as the equivalent of Départements (Departments) in Cote D'Ivoire, Cercles (Circles) in Mali, Départements (Departments) in Senegal, Sectors in Guinea-Bissau, and Divisions in Gambia. Population data [LandScan (2012)], was obtained from Oak Ridge National Laboratory (http://web.ornl.gov/sci/landscan/). ArcGIS v.10.2 (ESRI, Redlands CA) was used to calculate the population-weighted centroids for each district (or equivalent) and computing the geodesic distances between each district to all others. The population-weighted centroid was the center of a given area, adjusted for the density of the population within that area.

Technical Appendix Figure 1 shows the combination of the Geospatial data and the Case Counts Data (aggregated over 3 week periods for a simpler illustration), using data available through Week 39 [September 27]. At the time of our initial analysis (week-ending August 16, 2014, [epidemiological week 33]), the World Health Organization had reported 2,218, confirmed, probable and suspected cases in West Africa, with 523 in 14 of 34 districts in Guinea, 849 cases in 13 of 14 districts in Sierra Leone, and 846 cases among 12 of 15 districts in Liberia.

Calculations

Inverse Distances:

Sum of Inverse Distances (SID), Nonweighted:

Let X_i (i= 1 to n) be the set of unaffected districts at time t.

Let Y_j (j= 1 to N) be the set of affected districts at time t.

$$\text{SID }(X_i)_t = \sum_{j=1}^{N} 1 / D_{(Xi \rightarrow Yj)}$$

Where $D_{(Xi \rightarrow Yj)}$ is the population centroid-based geodesic distance between X_i to Y_j.

Weighted SID (wSID) by the rolling sum on new cases counts in the past three weeks:

Let $X_{(1 \text{ to } i)}$ be the set of unaffected districts at time t.

Let $Y_{(1 \text{ to } j)}$ be the set of affected districts at time t.

Let C_j be the number of new cases in the past three weeks (i.e. weeks t-3,t-2 and t-1) in district Y_j where $C_j \geq 1$.

$$wSID\ (X_i)_t = \sum_{j=1}^{N} C_j / D_{(Xi \rightarrow Yj)}$$

Where $D_{(Xi \rightarrow Yj)}$ is the population centroid-based geodesic distance between X_i to Y_j. $C_j \geq 1$ was used to prevent multiplication by zero (i.e. versus using where $C_j > 0$) and is justified by the results of Model 1 (see Technical Appendix Table 2). Model 1, which is statistically significant and uses a Nonweighted SID shows that all affected districts still have some influence on the probability of nonaffected districts becoming affected regardless of the number of new cases they have reported in the preceding three weeks

Goodness of Fit and Correlation:

We measured goodness of fit of the models to the data available through week 33 by assessing how well the models agreed with the set of districts reporting being affected at the time of analysis. To do this, we computed the predicted probability ρ_i of an individual district *i* being affected at each outbreak week (see Individual District Probabilities section below). Then we calculated the log likelihood (LL) for the set of districts already affected:

$$LL = \sum_{i} I_i * \log(p_i) + (1 - I_i) * \log(1 - p_i)$$

Where I_i =1 if the district was officially affected and $I_i = 0$ when nonaffected. The larger the LL the better the fit.

We also computed a Partial Correlation coefficient for each model. This was the marginal contribution of a single predictor to reducing the unexplained variation in affected/nonaffected outcome. The partial correlation indicates the explanatory value attributable to the predictor.

All analyses were completed using SAS for Windows version 9.3 (SAS Institute Inc., Cary, NC, USA).

Model Comparisons

Technical Appendix Table 2 shows the results of the various models which were evaluated for their ability to explain the regional spread of Ebola. All of the models were statistically significant at the .0001 level (Wald Chi-Square test) when testing the joint effect of the predictors included in the model. Model 3 fit the data the best (i.e. largest LL) and had the best explanatory value [Partial Correlation total > 60%].

The LL measure is criticized because it does not take into account the number of parameters used in the model, however, when the models were evaluated using the Akaki Information Criteria (AIC), which explicitly does so, Model 3 was still the best fitting model: Model 1= 307.0; Model 2=292.6; Model 3=283.7.

Reporting Delay Analysis:

Several districts reported a relatively high number of cases in the week in which they first reported having cases. We considered reporting delays as a possible reason for the high count (under the assumption that a portion of these cases should have been reported in the prior week) and conducted an analysis to examine the impact on our results. In this analysis we adjusted the number of cases at the week of first report when it was greater than 10 [5 occurrences out of the 39 districts having reported cases by week 39] by distributing the case count over two weeks: one half remained in the same week and the other was assigned to the prior week. Technical Appendix Table 2 shows the results of this analysis. Models 1 and 4 saw slight improvement in their goodness of fit, but in the better fitting models [2 and 3], there was no improvement seen.

Internal Validation:

We randomly eliminated 10% (n=6) of districts from the outbreak data available through week 33 and fit Model 3 to this dataset. Afterwards, we examined whether the predicted individual probability of becoming affected for the eliminated districts (calculated using the original model with the full complement of data) fell within the 95% confidence interval generated by the model without these districts. We found that all probabilities fell within the 95% CI ranges, suggesting that the model based on the full dataset was appropriate.

Individual District Probabilities

In order to calculate the individual probabilities of a particular district becoming affected we used the maximum likelihood (ML) method to fit Model 3 to the outbreak data and obtain estimate values for the parameters α, β_1 and β_2. The results of the initial ML estimation based on data available through week 33 are presented in Technical Appendix Table 3. Technical Appendix Figure 2 shows the resulting relationship between the predictors in Model 3 and the probability of becoming affected (also based on data through week 33).

As the outbreak continued, Model 3 was refit (i.e. new parameter values obtained for α and β) to the data at week 36, and again at weeks 39 and 42, in order to update the predicted probabilities for individual districts (Figures 1 and 2). The updated fit at week 42 was used to calculate the probabilities

for districts among the four countries bordering those primarily affected by the Ebola outbreak (Figure 2).

Additional Results

After determining the wSID parameter was significant in all main models we examined the differences between the wSID between affected and unaffected districts at each outbreak week. These results are shown in Technical Appendix Figure 3A. A discernable difference between the average wSID among affected and unaffected districts is apparent at weeks 29 through 33, but not in prior outbreak weeks. This may suggest that the relationship between distance and the probability of being affected only becomes influential at some threshold: At week 29 the difference in the overall wSID for districts that became affected that week and those that did not was 0.00035. Additionally, when we examined the differences between the average wSID in affected and unaffected districts within each country individually we did not observe a similar pattern for weeks 29 through 31 (Technical Appendix Figure 3B). This corroborates the lesser fit of Model 4 Reduced (Technical Appendix Table 2), which examined each country individually. It also suggests that border closings have not had a great deal of impact in slowing the cross-border spread of disease; in-line with our assumption that borders were porous.

Technical Appendix Table 1. Data definitions used for special data scenarios

Data scenario	Week definition	Case count weighting
Districts initially reporting suspect cases only and which then reported 0 cumulative cases in a later week due to invalidation of suspect cases through laboratory testing	We used the most recent week in the outbreak after which the cumulative case count remained above 0 as the week in which the district became affected.	Weekly counts comprised of suspect cases which were later invalidated were changed to 0 values
Districts with gaps in reporting	We assumed that there was no reversion back to unaffected during the weeks with absent data. We used the earliest week with reported cases as the week in which the district became affected.	During reporting gap weeks we used the number of cases reported in the week just prior to the reporting gap.
Affected districts with no new cases in a particular week	NA	Case count weighting = 1 (otherwise the inverse distance of that district would not contribute to the wSID)

Technical Appendix Table 2. Summary of models used to characterize the spread of Ebola to nonaffected districts - analysis completed on data available through week 33 (August 16, 2014)

Model	Probability of becoming affected	Log-likelihood (LL) No adjustment	Log-likelihood (LL) Reporting Adjusted†	Partial Correlation*
Model 0 (Intercept only)	$1/1 + e^{-(\alpha)}$	–165.03 (ns)	–165.03 (ns)	Not applicable
Model 1 (SID)	$1/1 + e^{-(\alpha + \beta 1SID)}$	–159.49	–151.62	29.2
Model 2 (wSID)	$1/1 + e^{-(\alpha + \beta 1(wSID))}$	–144.29	–144.33	36.0
Model 3 (wSID + Population)	$1/1 + e^{-(\alpha + \beta 1(wSID) + \beta 2(Population))}$	**–138.85**	**–139.19**	**60.6**
Model 4‡ ($wSID_G$ + $wSID_L$ + $wSID_S$)	$1/1 + e^{-(\alpha + \beta 1(wSIDG) + \beta 2(wSIDL) + \beta 3(wSIDS))}$	–151.06	–139.64	27.0
Model 4 Reduced‡<br>a) only Guinea data ($wSID_G$)<br>b) only Sierra Leone data ($wSID_S$)<br>c) only Liberia data ($wSID_L$)	$1/1 + e^{-(\alpha + \beta x(wSIDy))}$<br>Where $\beta_x wSID_y$ = $\beta 1(wSID_G)$, $\beta 2(wSID_L)$, or $\beta 3(wSID_S)$<br>From Model 4's equation above	a) –65.3 (ns)<br>b) –41.5<br>c) –47.6 (ns)	Not evaluated	Not evaluated

*ns, the difference between the model with covariates and the model with the intercept only was not statistically significant at the .0001 level.
* Partial correlations were calculated for the model with "No adjustment" for case reporting delays. For models with more than one parameter [Models 3 and 4] the value shown is the sum of the partial correlation coefficients as follows. Model 3; wSID=37.9 and Population=22.7. Model 4; $wSID_G$=0.1, $wSID_L$=–0.1, $wSID_S$=27.0.
† When the number of cases at the week of first report was greater than 10 we halved the number of cases at first report and made the week of first report 1 week earlier, by assigning half the cases to that week.
‡ Subscripts G, S, and L correspond to Guinea, Sierra Leone, and Liberia, respectively.

Technical Appendix Table 3. Results of maximum likelihood estimation for Model 3; completed on data available through week 33 (August 16, 2014)

Parameter	Symbol	Estimate	Standard Error	Wald Chi-Square	Significance Level	Partial Correlation
Intercept	α	-5.1556	0.3950	170.3755	<.0001	
wSID	β_1	1794.8	255.4	49.3937	<.0001	0.3789
Population	β_2	2.628E-6	6.041E-7	18.9282	<.0001	0.2265

References

1. World Health Organization. Situation reports with epidemiological data: archive, 2014 Oct 20 [cited 2014 Aug 11]. http://www.who.int/csr/disease/ebola/situation-reports/archive/en/

2. Boender GJ, van den Hengel R, van Roermund HJ, Hagenaars TJ. The influence of between-farm distance and farm size on the spread of classical swine fever during the 1997–1998 epidemic in The Netherlands. PLoS ONE. 2014;9:e95278. PubMed http://dx.doi.org/10.1371/journal.pone.0095278

3. Cauchemez S, Ledrans M, Poletto C, Quenel P, de Valk H, Colizza V, et al. Local and regional spread of chikungunya fever in the Americas. Euro Surveill. 2014;19:20854 [cited 2014 Aug 11]. Epub. http://www.eurosurveillance.org/Public/Articles/Archives.aspx

4. Johansson MA, Powers AM, Pesik N, Cohen NJ, Staples JE. Nowcasting the spread of chikungunya virus in the Americas. PLoS One. 2014;9(8):e104915 [cited 2014 Aug 11]. Epub. http://www.plosone.org/article/info%3Adoi%2F10.1371%2Fjournal.pone.0104915

5. World Health Organization Ebola Response Team. Ebola virus disease in West Africa—the first 9 months of the epidemic and forward projections. N Engl J Med. 2014;371:1481–95 [cited 2014 Oct 20]. Epub. http://www.nejm.org/doi/full/10.1056/NEJMoa1411100

6. Fofana U. Sierra Leone shuts borders, closes schools to fight Ebola. Reuters; 2014 Jun 11 [cited 2014 Oct 3]. http://uk.reuters.com/article/2014/06/11/us-health-ebola-leone-idUKKBN0EM2CG20140611

7. Ebola crisis: Guinea closes borders with Sierra Leone and Liberia. The Guardian; 2014 Aug 9 [cited 2014 Oct 3]. http://www.theguardian.com/society/2014/aug/09/ebola-guinea-sierra-leone-liberia

8. Christensen J. Liberia closes its borders to stop Ebola. CNN; 2014, Aug 22 [cited 2014 Oct 3]. http://www.cnn.com/2014/07/28/health/ebola-outbreak-west-africa/

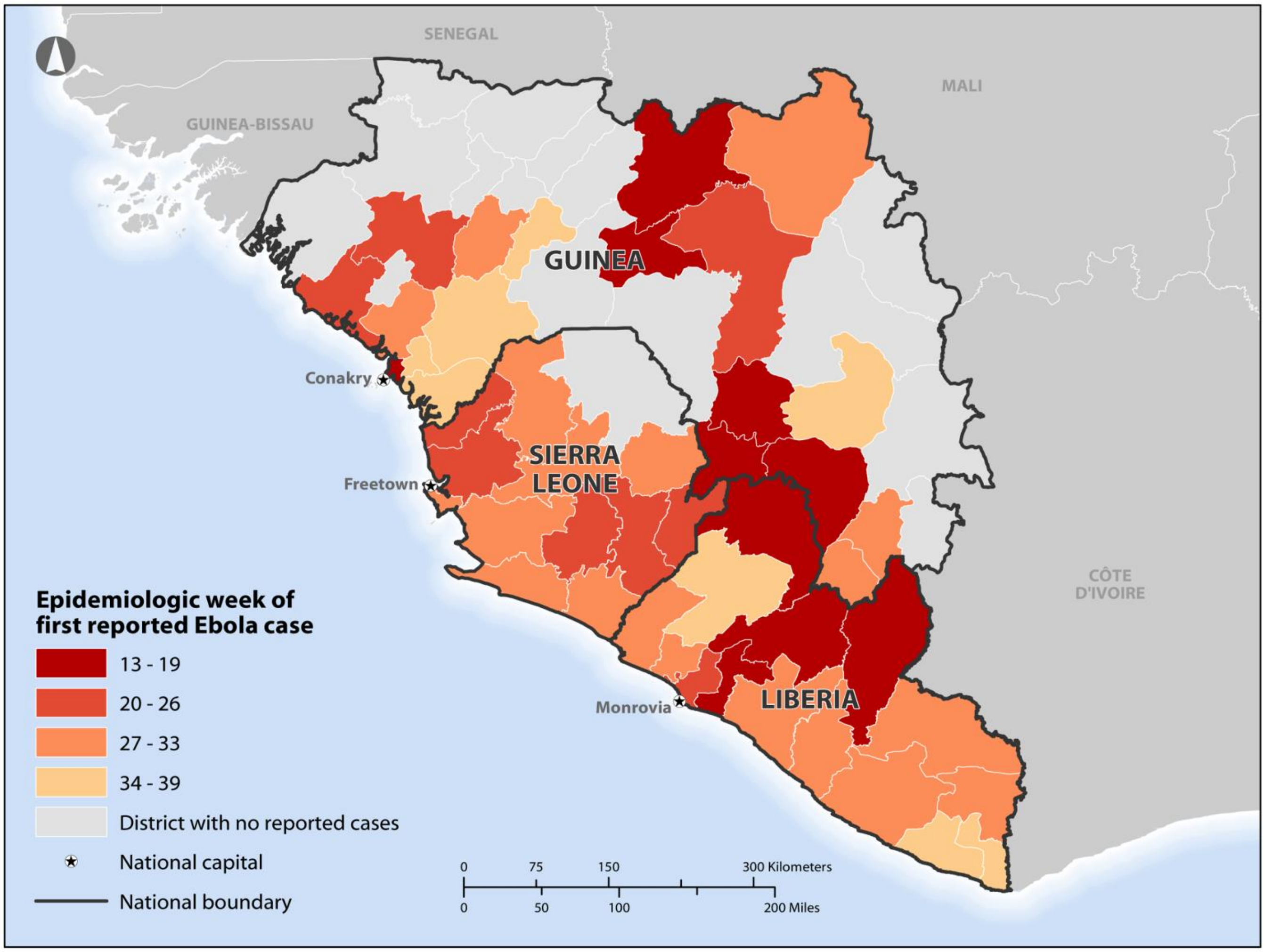

Technical Appendix Figure 1. Map of the primary Ebola affected countries by week districts became affected (using data available through week 39 [September 27] (*2*) Notes: International borders were closed as follows: Sierra Leone, June 11th [week 24]; Guinea, August 9th [week 32]; Liberia, August 22 [week 34] (*6*–*8*).

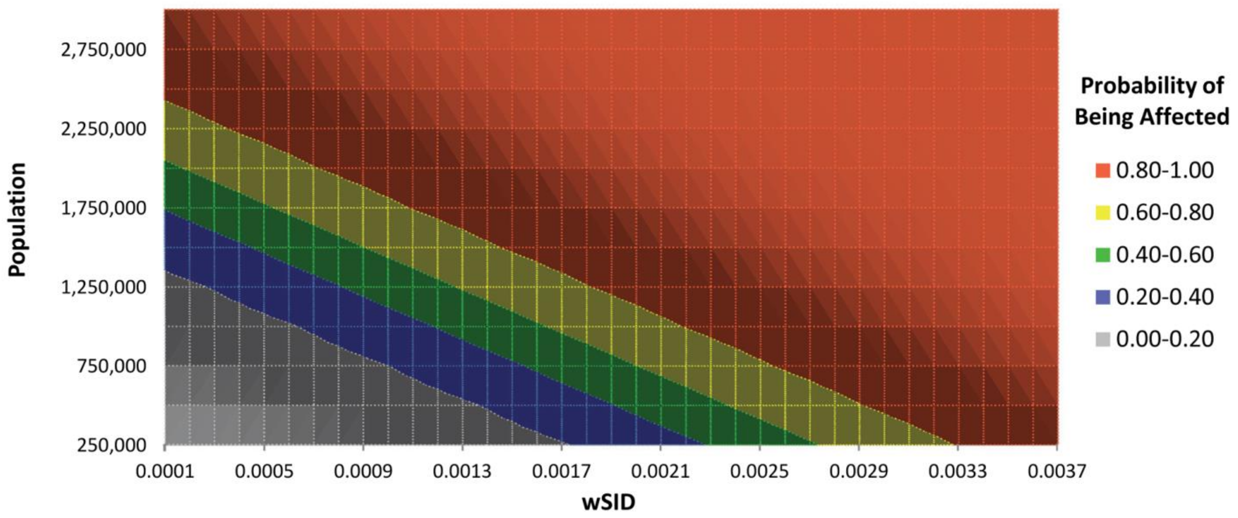

Technical Appendix Figure 2. Predicted probability of a district becoming affected as a function of its population and the weighted-SID to affected areas (fitted to data available through week 33 [August 16])

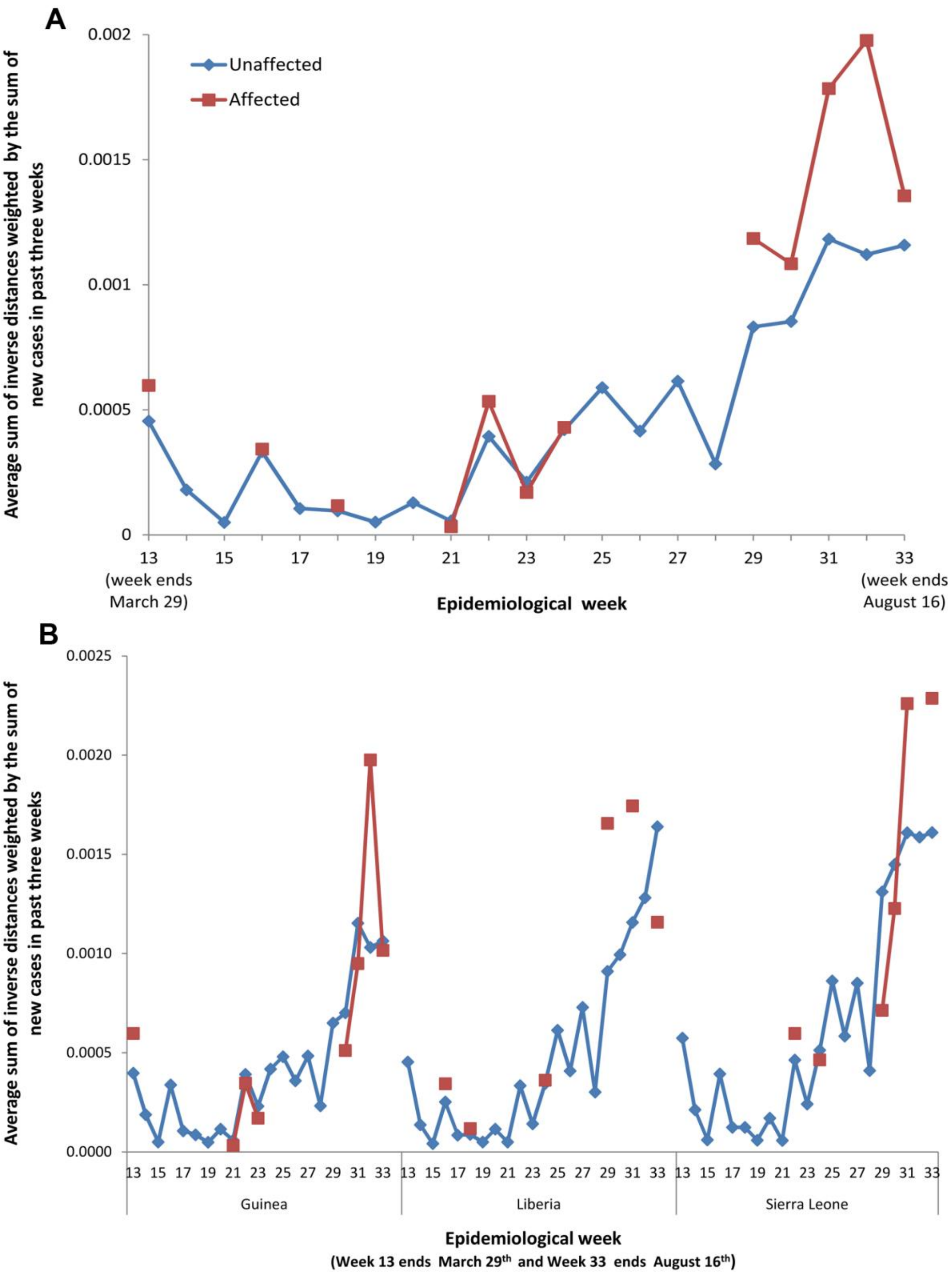

Technical Appendix Figure 3. Average weighted-SID by outbreak week and affected status for all primarily-affected countries (Panel A) and for each individual country (Panel B) Notes: The average of the weighted-SID's influence (wSID) on districts that were affected (red-square marker) is compared here to the average (wSID) for nonaffected districts (blue diamond marker) at each outbreak week. Districts became affected on just 12 of the outbreak weeks, resulting in the gaps in the affected line (as of week 33, when this analysis was completed). A discernable difference between the average wSID among affected and nonaffected districts is apparent when all countries are shown together at weeks 29 through 33 (Panel A).
